# Microscopy-Based Automated Live Cell Screening for Small Molecules That Affect Ciliation

**DOI:** 10.3389/fgene.2019.00075

**Published:** 2019-02-12

**Authors:** Peishan Zhang, Anna A. Kiseleva, Vladislav Korobeynikov, Hanqing Liu, Margret B. Einarson, Erica A. Golemis

**Affiliations:** ^1^School of Pharmacy, Jiangsu University, Zhenjiang, China; ^2^Molecular Therapeutics Program, Fox Chase Cancer Center, Philadelphia, PA, United States; ^3^Department of Biochemistry and Biotechnology, Kazan Federal University, Kazan, Russia; ^4^Department of Pathology and Cell Biology, Columbia University, New York, NY, United States

**Keywords:** ciliary disassembly, high content imaging, drugs, ADPKD, targeted therapy, aurora kinase A, heat shock protein 90, screening

## Abstract

The primary monocilium, or cilium, is a single antenna-like organelle that protrudes from the surface of most mammalian cell types, and serves as a signaling hub. Mutations of cilia-associated genes result in severe genetic disorders termed ciliopathies. Among these, the most common is autosomal dominant polycystic kidney disease (ADPKD); less common genetic diseases include Bardet–Biedl syndrome, Joubert syndrome, nephronophthisis, and others. Important signaling cascades with receptor systems localized exclusively or in part at cilia include Sonic Hedgehog (SHH), platelet derived growth factor alpha (PDGFRα), WNTs, polycystins, and others. Changes in ciliation during development or in pathological conditions such as cancer impacts signaling by these proteins. Notably, ciliation status of cells is coupled closely to the cell cycle, with cilia protruding in quiescent (G0) or early G1 cells, declining in S/G2, and absent in M phase, and has been proposed to contribute to cell cycle regulation. Because of this complex biology, the elaborate machinery regulating ciliary assembly and disassembly receives input from many cellular proteins relevant to cell cycle control, development, and oncogenic transformation, making study of genetic factors and drugs influencing ciliation of high interest. One of the most effective tools to investigate the dynamics of the cilia under different conditions is the imaging of live cells. However, developing assays to observe the primary cilium in real time can be challenging, and requires a consideration of multiple details related to the cilia biology. With the dual goals of identifying small molecules that may have beneficial activity through action on human diseases, and of identifying ciliary activities of existing agents that are in common use or development, we here describe creation and evaluation of three autofluorescent cell lines derived from the immortalized retinal pigmented epithelium parental cell line hTERT-RPE1. These cell lines stably express the ciliary-targeted fluorescent proteins L13-Arl13bGFP, pEGFP-mSmo, and tdTomato-MCHR1-N-10. We then describe methods for use of these cell lines in high throughput screening of libraries of small molecule compounds to identify positive and negative regulators of ciliary disassembly.

## Introduction

Primary cilia, also known as monocilia, are evolutionarily conserved, antenna-like organelles that protrude from the cell surface of almost all types of mammalian cell types, with limited exceptions (e.g., lymphocytes). Ciliated cells were a topic of considerable interest as early as the first half of the 19th century (Sharpey, 1835). For much of the first part of the 20th century, cilia were considered vestigial organelles, of unclear functionality. Starting in 1960s, a growing number of studies noted variations in ciliation across distinct cell types, and in relationship to proliferative state of cells. These studies led to a focus on the machinery governing ciliation, and to investigation of whether ciliary state in turn could reciprocally affect proliferation or other aspects of cell signaling. Early and ongoing studies have defined the cilium as an organelle important for mechano- and chemosensation, with mechanisms of signal transduction in some cases well defined, and in other cases still unclear (Seeger-Nukpezah and Golemis, 2012; Delling et al., 2016; Nag and Resnick, 2017; Spasic and Jacobs, 2017). Signaling receptors concentrated or exclusively localized to cilia make this organelle an important platform for the signal transduction, associated with Hedgehog (HH), WNT, Notch, and platelet derived growth factor alpha (PDGFRα) signaling pathways (Goetz et al., 2009; Liu et al., 2018), among others.

Detailed studies of ciliation patterns led to the recognition that this organelle undergoes assembly and disassembly dependent on cell cycle phase. Mammalian cells undergoing mitosis are invariably unciliated. Assembly of the primary cilium initiates immediately after mitotic exit in early G1 phase, and is most notable in cells that become quiescent, entering G0. Ciliary disassembly or resorption commonly occurs in S or G2 phase, although there is variation dependent on specific cell backgrounds. This timing of ciliary assembly/protrusion and disassembly/resorption in part reflects the fact that the ciliary structure emanates from a basal body, that nucleates the nine paired microtubular bundles that constitute the core of the central ciliary axoneme. The basal body is derived from the mother centriole of the centriolar pair present in the cell following cytokinesis, based on a process of differentiation. In the early stages of ciliary assembly, Golgi-derived vesicles localize to the vicinity of the mother centriole, which associates with specific accessory proteins that form distal appendages associated with the nascent basal body. The recruitment of targeted vesicles contributes to the formation of new membrane and transport of ciliary localized proteins, organized around the axonemal shaft. Specific members of the intraflagellar transport (IFT) particle protein family traffic ciliary components along the axoneme, elongating the axoneme itself and concentrating specific cilia-associated proteins on the membrane of the mature cilium (Pedersen et al., 2008). This process is reversed during ciliary disassembly, releasing the basal body from distal appendages and other cilia-specific proteins. The centrioles then undergo a discrete differentiation process involving association with a distinct group of proteins that allow it to act as a centrosome, duplicate, nucleate cytoplasmic microtubules and signaling complexes during interphase, and act as a microtubule organizing center (MTOC) that anchors the bipolar spindle during mitosis.

**FIGURE 1:**
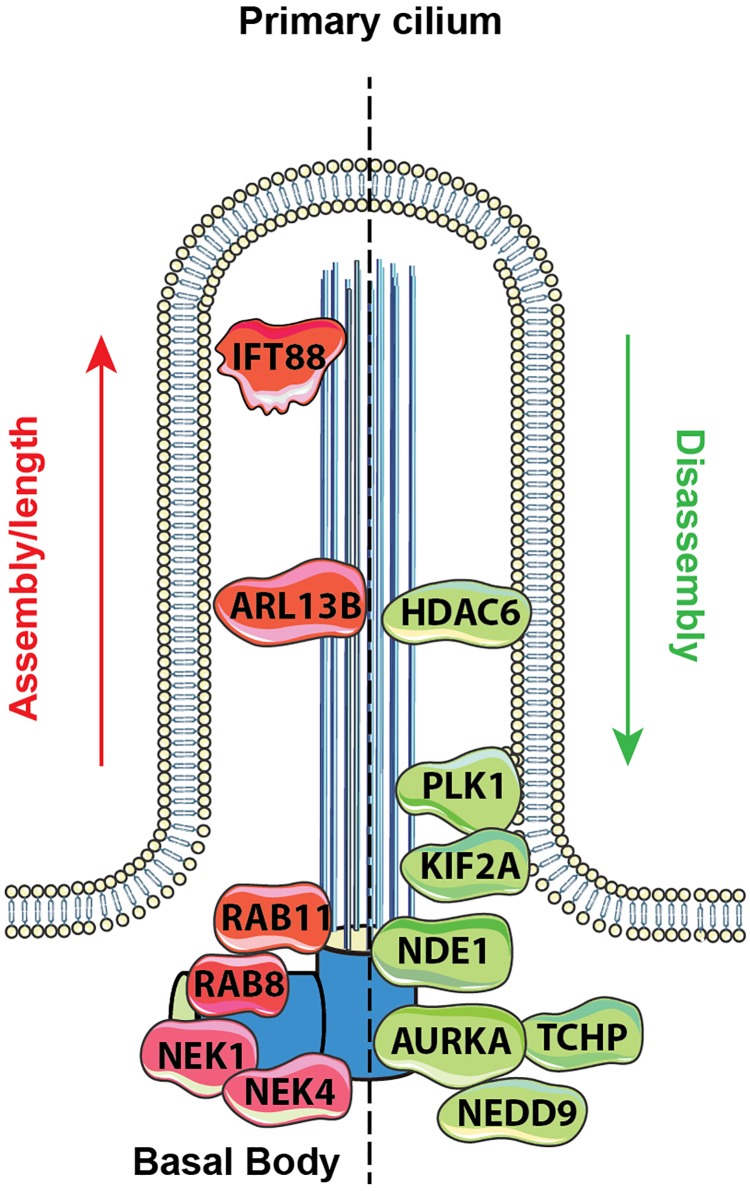
Proteins regulating ciliary assembly and disassembly. Mechanisms of ciliary dynamics are complex and require the orchestrated action of numerous proteins. Some examples of proteins regulating ciliary protrusion (red) and resorption (green) are shown. The Rab GTPases (e.g., Rab8 and Rab11) are active at the early stages of cilia formation, promoting targeted vesicular trafficking required for the primary ciliogenesis (Knodler et al., 2010). Assembly of the primary cilium is highly dependent on the proper functioning of the IFT – a bidirectional movement throughout the axonemal microtubules which is essential for cilium elongation and maintenance. One of the key proteins controlling IFT-dependent ciliogenesis is IFT protein 88 homolog (IFT88); mutations inactivating IFT88 to an abnormally short cilia phenotype and development of polycystic kidney disease (Pazour et al., 2000). The movement of IFT particles along the microtubules is supported by a group of microtubule-based motors, which include kinesin family proteins (KIFs), providing anterograde transport (toward the cilia tip) and dynein family proteins (DYNs), providing retrograde transport (toward the cilia base). Cilia formation is also controlled by NIMA-related kinases (NRKs), namely, Nek1 and Nek4, which are often determined as mitotic kinases (Mahjoub et al., 2005; Coene et al., 2011). Additionally, ciliogenesis is regulated by small GTPases, including Arl13b, which localizes exclusively to the primary cilium, where it stabilizes the association between IFT-A and IFT-B complexes (Li et al., 2010).

Because of the need to exactly synchronize ciliary assembly/disassembly cycles with cell cycle, the process is highly regulated through the activity of protein kinases and other enzymes that modify the IFT proteins, the axonemal microtubules, the cargo to be transported to the cilia, and other relevant proteins. Mechanistic details of these processes have been extensively reviewed (Cao et al., 2009; Ishikawa and Marshall, 2017; Korobeynikov et al., 2017; Nachury, 2018). Additional sources of regulation relevant to cilia include control of ciliary length, which is partially but not completely linked to cell cycle status, and can influence the strength of response to extracellular stimuli with ciliary receptor systems (Chien et al., 2018; Drummond et al., 2018). Definition of the cellular signals regulating ciliation has been a topic of much interest. Activation of the Aurora-A (AURKA) kinase has been shown to be a proximal signal for ciliary disassembly, based on interactions with its partners HEF1/NEDD9, and other proteins (Pugacheva and Golemis, 2006; Pugacheva et al., 2007; Plotnikova et al., 2012). As cells emerge from G0/quiescence in response to the addition of growth factors, multiple upstream signaling systems transiently activate AURKA at the basal body; conversely, a separate set of signaling proteins restrains AURKA activity, and collaborates to promote ciliary protrusion, following cytokinesis (reviewed in Korobeynikov et al., 2017). As the knowledge of which proteins regulate ciliary formation and resorption has gradually become more detailed, it has become apparent that many of the proteins involved were first defined based on roles in other biological processes (Figure 1).

Disassembly of the primary cilium is associated with the cell cycle and starts when cells exit G1/G0 phase. At the early stages, in a response to growth factors stimuli, the disassembly of the primary cilium is associated with the de-polymerization of the axoneme microtubules, which is partially controlled by an activation of kinesin superfamily protein 2A (KIF2A) and polo-like kinase-1 (PLK1) (Seeger-Nukpezah et al., 2012; Miyamoto et al., 2015). Another key event in cilium disassembly is the activation of Aurora A kinase (AURKA) at the basal body by a scaffolding protein NEDD9, which in turn activates HDAC6 and promotes de-acetylation of the axoneme tubulins with following destabilization of the axoneme microtubules (Pugacheva et al., 2007). Activation of AURKA in disassembly is also promoted by trichoplein (TCHP; Inoko et al., 2012) and other proteins (Korobeynikov et al., 2017). Some of these proteins act continuously to inhibit cilia assembly during cell cycle, including TCHP and nuclear distribution gene E homolog 1 (NDE1); the latter is expressed at high levels during mitosis and suppresses retrograde IFT complexes movement to suppress cilia formation (Kim et al., 2011).

Further ciliary regulatory systems control the specific localization of components of discrete signaling systems to the cilia, which can profoundly influence the activity of signaling pathways. In one of the best studied examples, the extracellular factor Sonic Hedgehog (SHH) binds to a ciliary localized receptor, Patched (PTCH1). In the absence of SHH binding, ciliary PTCH1 normally represses the activity of a downstream signaling cascade dependent on the GLI family of transcription factors; however, following SHH-PTCH1 interactions at cilia, PTCH1 translocates from the cilium, and Smoothened (SMO) enters, where it interacts with and activates GLI transcription factors (Bangs and Anderson, 2017). Because of this and other ciliary signaling receptor systems, control of ciliation is relevant to a number of clinically significant human pathological conditions (Pan et al., 2013).

Ciliopathies comprise a group of disorders associated with genetic mutations causing defects in ciliary structure or signaling, affect a significant number of individuals. Over 35 types of ciliopathy have been defined (Reiter and Leroux, 2017). The most common ciliopathy, autosomal dominant polycystic kidney disease (ADPKD), affects 1 in 500 people, and currently lacks effective treatments. Recent developments raise the possibility that targeting ciliation may be useful in slowing the course or alleviating the symptoms of at least some forms of this disease (Ma et al., 2013; Nikonova et al., 2018). Other less frequent ciliopathies associated with severe symptoms include Bardet–Biedl syndrome (BBS; Zaghloul and Katsanis, 2009), Meckel–Gruber syndrome, Joubert syndrome, and others (Mitchison and Valente, 2017); these also would benefit from better knowledge of, and tools to manipulate, ciliation. In other conditions, such as cancer, manipulation of ciliation has the potential to disrupt or intensify signaling axes relevant to disease formation, progression, and response to treatment (discussed extensively in Liu et al., 2018).

Of the mammalian proteins defined as regulating ciliation, a number have been shown to have causative or pro-oncogenic roles in cancer, and in some cases, targeted drugs designed to inhibit their activity have been developed. Notably, some of these drugs have been found to strongly influence either ciliation, or the trafficking of cellular signaling proteins to cilia. For example, the AURKA inhibitor alisertib increases cilia length and prevents ciliary resorption *in vitro* and *in vivo* (Pugacheva et al., 2007; Nikonova et al., 2014). Conversely, ganetespib, an inhibitor of heat shock protein 90 (HSP90) inhibits proteasomal degradation of NEK8 and the AURKA activator trichoplein, causing AURKA activation and promoting loss of ciliation, *in vitro* and *in vivo* (Seeger-Nukpezah et al., 2013; Nikonova et al., 2018). The control of ciliary dynamics remains far from completely defined; surprisingly, a recent study screening 1600 small molecule compounds in a human pancreatic cell line, CFPAC-1, identified 118 cilium-enhancing compounds for which no prior activity at cilia had been identified (Khan et al., 2016), suggesting modulation of ciliation status may not be an uncommon on-target or off-target effect of drugs of clinical interest. If so, it is considerable interest to be able to identify such compounds efficiently, as they may have unexpected “off-target” activities based on control of ciliary signaling systems such as SHH, which has important autocrine signaling in some cell types, and also plays an important role in paracrine signaling between various cell types, in both normal and pathogenic growth conditions (Lee et al., 2014; Tape et al., 2016; Bangs and Anderson, 2017). In one example particularly relevant to ciliopathies, treatment of a mouse model for ADPKD with an AURKA inhibitor under evaluation in the clinic blocked ciliary disassembly and significantly exacerbated disease symptoms (Nikonova et al., 2014), emphasizing the potential risks of perturbing ciliation with such genetic disorders.

There are many model systems that have been used for screening to detect modifiers of ciliation. Over the past 40 years, genetic and biochemical experiments performed in the unicellular alga *Chlamydomonas reinhardi* (Lefebvre and Rosenbaum, 1986), the nematode *Caenorhabditis elegans* (Muller et al., 2011), in *Danio rerio* (zebrafish) (Malicki et al., 2011), and others (Vincensini et al., 2011) have yielded critical information about genes regulating ciliary formation and length control. Our focus here is on the evaluation of small molecule agents relevant to humans and potentially other mammalian cancer models. For this purpose, to avoid potentially misleading results arising from imperfect conservation of drug targets across large evolutionary distances, it is optimal to develop a screening system based on the use of cultured cell lines. Cell lines that have been extensively exploited in studies of ciliation include hTERT1-immortalized human retinal pigmented epithelium cells (hTERT-RPE1 cells) (Bodnar et al., 1998), murine NIH3T3 fibroblasts, the murine inner medullary collecting duct cell line model (mIMCD3), and epithelial kidney cells.

We here describe a microscopy-based screening method that can be applied in high throughput to identify small molecules which affect ciliation. Numerous microscopic approaches are effective in low to moderate throughput for evaluating ciliation and ciliary dynamics in living or fixed cells, including differential interference contrast (DIC) microscopy, or confocal imaging of immunostained cilia. To minimize manipulation of cells and facilitate high throughput assessments, this procedure is based on the use of cell models stably expressing fluorescent proteins (e.g., EGFP, TdTomato) targeted to the cilia by fusion to a cilia-targeting moiety. We present data comparing the efficiency of visualization of cilia using targeting moieties provided by fusion of these fluorescent moieties to ADP-ribosylation factor-like protein 13b (ARL13b), SMO, and melanin-concentrating hormone receptor 1 (MCHR1) in the hTERT1-RPE1 cell line. We discuss relevant issues for optimizing analysis, and present an approach using an automated imaging system to visualize, quantify, and establish significance of ciliation data, using alisertib and ganetespib as examples of small molecules that influence ciliation.

## Materials and Methods

### Development and Culture of Cell Models

#### Introduction, hTERT-RPE1 Cells

Although many cell lines could potentially be used for high throughput study of ciliation, a number of features have caused the hTERT-RPE1 (American Type Culture Collection, Manassas, VA, United States) (ATCC CRL4000) cell line to be a preferred model. hTERT-RPE1 cells were derived by transfecting primary human retinal pigmented epithelium cells with the catalytic component of human telomerase (hTERT; Bodnar et al., 1998). These cells are routinely maintained in DMEM/F-12 medium (Life Technologies, Carlsbad, CA, United States) with 10% FBS plus 1× penicillin/streptomycin, 1× Glutamax-I (Gibco/Thermo Fisher Scientific, Waltham, MA, United States), and 0.01 mg/ml hygromycin B (Life Technologies, Carlsbad, CA, United States). Using optimized culture conditions, it is possible to achieve ciliation of the substantial majority of the hTERT-RPE1 population (80–90% of cells), in contrast to other cell lines such as NIH3T3 in which maximum levels are closer to 60–70%. hTERT-RPE1 cells have a flat, spreading growth habit, and do not clump, which has supported their use not only for studies of ciliation, but also for studies of cell division and other processes requiring high resolution microscopy (e.g., Uetake and Sluder, 2004).

Although all protocols for generating cilia involve culture of cells at high density under conditions of little or no serum to induce quiescence, different cell lines become optimally ciliated under distinct growth regimens. In hTERT-RPE1 cells, for optimal induction of ciliation, cells are typically plated at 50–70% confluency, then maintained in serum free medium for 24–72 h. As a note of relevance to screening for regulation of ciliation, the longer cells are maintained under high density/low serum conditions, the longer it takes for them to respond to stimuli for ciliary resorption. With the goal of achieving maximally reproducible results, it is important to identify a single seeding density and timing for serum deprivation, and use this consistently.

#### Generation of Stable EGFP-Arl13b, EGFP-Smo, tdTomato-MCHR1 Expressing hTERT-RPE1 Cell Lines

A standard means of measuring ciliation is to fix cells with 4% paraformaldehyde (PFA), then use an antibody-based immunofluorescence approach to identify to proteins such as acetylated α-tubulin (Piperno and Fuller, 1985) to visualize the axoneme, in conjunction with an antibody to γ-tubulin (Muresan et al., 1993) to visualize the basal body. While this approach yields robust and highly quantifiable data, the need for fixation and staining with multiple antibodies results in slower throughput and higher costs than use of cilia-localizing fluorescent proteins. In this study, we have comparatively evaluated suitability of three fluorescent ciliary proteins, based on ciliary localization provided by ARL13b, SMO, and MCHR1 and autofluorescence provided by fused EGFP and tdTomato moieties.

#### Rationale for Choosing Arl13b, SMO, and MCHR1 as the Target Genes

**FIGURE 2:**
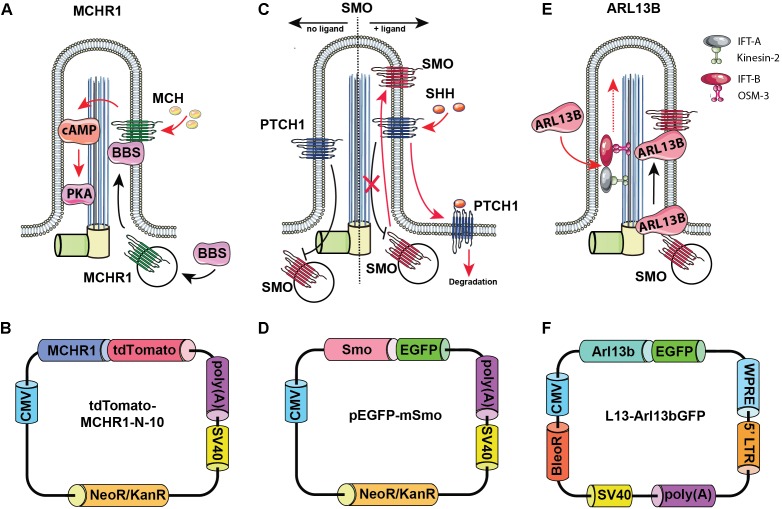
Rationale for choosing Arl13b, SMO, and melanin-concentrating hormone receptor 1 (MCHR1) as cilia-targeting moieties. Mechanism of MCHR1 action **(A)** and a schematic **(B)** for tdTomato-MCHR1-N-10 plasmid. MCHR1 is a protein which belongs to a class A of G-protein-coupled receptors (GPCRs) and localizes in neuronal cilia in the hypothalamus (Green and Mykytyn, 2010). The translocation of MCHR1 to the ciliary membrane by the BBSome complex (Nachury, 2018) allows it to play an important role in controlling energy homeostasis potentially through modulating cAMP signaling (Schou et al., 2015). Mechanism of SMO action **(C)** and a schematic **(D)** for pEGFP-mSmo plasmid. Hedgehog (HH) pathway is uniquely associated with the primary cilium. Two key receptors for this pathway – Smoothened (SMO) and Patched (PTCH1) – localize within the ciliary membrane depending on the pathway’s activation stage. In the absence of HH ligands, PTCH1 accumulates at the ciliary membrane preventing translocation of Smo to the cilia. When the ligand binds PTCH1, it leaves the cilia allowing SMO to translocate and activate the pathway. Mechanism of Arl13b action **(E)** and a schematic **(F)** for L13-Arl13bGFP plasmid. Arl13b is a small guanosine triphosphate (GTPase) which localizes exclusively to the primary cilium, where it controls ciliogenesis through stabilizing the coordination between IFT-A and IFT-B complexes (Li et al., 2010). Additionally, Arl13b regulates translocation of SMO receptor to the cilia (Larkins et al., 2011).

*Arl13b* is a small guanosine (Figure 2)triphosphate (GTPase) which localizes exclusively to the primary cilium, where it controls ciliogenesis through stabilizing the coordination between IFT-A and IFT-B complexes (Li et al., 2010). Additionally, Arl13b regulates translocation of SMO receptor to the cilia (Larkins et al., 2011).

As noted above, the HH pathway is uniquely associated with the primary cilium (Bangs and Anderson, 2017). In the absence of HH ligands, PTCH1 accumulates at the ciliary membrane, preventing translocation of SMO to the cilia. When the HH ligand binds PTCH1, PTCH1 leaves the cilia, allowing SMO to translocate and activate the pathway.

Melanin-concentrating hormone receptor 1 is a protein which belongs to a class A of G-protein-coupled receptors (GPCRs) and localizes in neuronal cilia in the hypothalamus, where it plays an important role in controlling energy homeostasis potentially through modulating cAMP signaling (Green and Mykytyn, 2010; Schou et al., 2015).

For each of these ciliary proteins, fusions to fluorescent proteins have been developed and are publicly available. The L13-Arl13bGFP (Larkins et al., 2011) (Addgene, plasmid #40879), pEGFP-mSMO (Chen et al., 2002) (Addgene, plasmid #25395), tdTomato-MCHR1-N-10 (Addgene, plasmid #58114; a gift of Michael Davidson) constructs, expressing the murine, full length coding sequences for Arl13b, SMO, and MCHR1 as fusions to small fluorescent proteins, were obtained from Addgene (Cambridge, MA, United States). hTERT-RPE1 cells with overexpression of tdTomato-MCHR1 and pEGFP-SMO were generated by transfection with the corresponding constructs using Lipofectamine 2000 transfection reagent (Life Technologies, Carlsbad, CA, United States), selection with G418 (500 μg/ml, for approximately 2 weeks), and further clonal selection by limiting dilution assay. Stable Arl13bGFP overexpression in the hTERT-RPE1 cell line was achieved via lentiviral delivery of L13-Arl13bGFP construct. Briefly, lentivirus was packaged by co-transfection of L13-Arl13bGFP construct with the packaging and envelope plasmids (psPAX2 and pMD2.G) by calcium phosphate transfection into HEK293T cells (Kingston et al., 2003). Medium was collected after 48 and 72 h, combined and filtered through a 0.45 μm filter, then applied to hTERT-RPE1 cells. At 72 h post-infection, hTERT-RPE1 cells were selected with 400 μg/ml zeocin for approximately 2 weeks and additionally selected by clonal selection by limiting dilution assay. All experiments with lentiviruses are conducted according to biosafety level 2 (BSL-2) guidelines.

**FIGURE 3:**
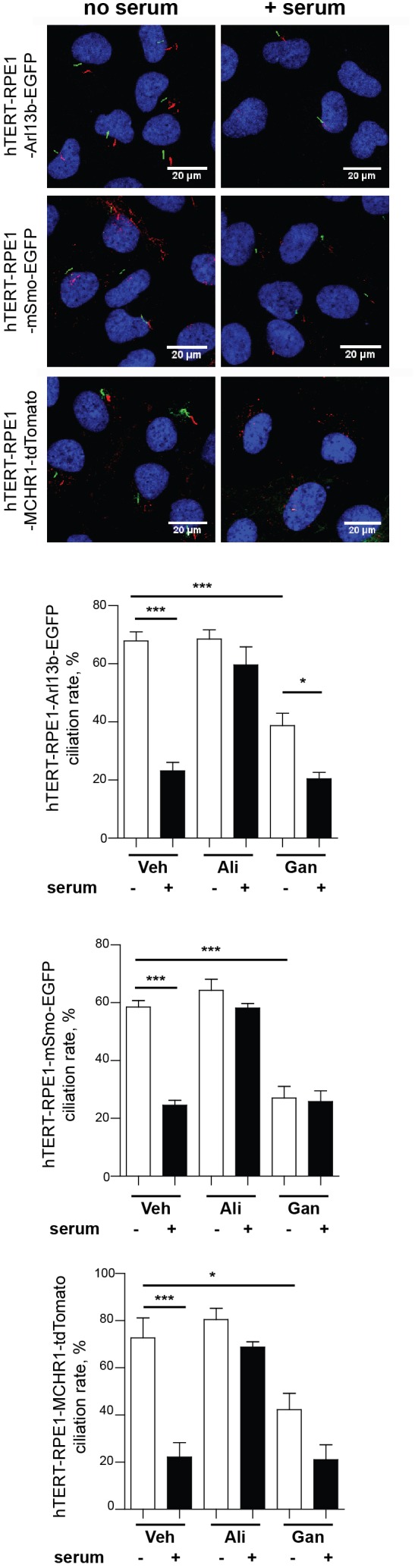
Confocal images of tTERT-RPE1 cells expressing L13-Arl13bGFP, pEGFP-mSMO, and tdTomato-MCHR1-N-10. Top, representative images of RPE1-Arl13b-EGFP (green) pEGFP-mSMO (green), and tdTomato-MCHR1-N-10 (red) in cells also visualized with antibody to acetylated a-tubulin (red with Arl13b and SMO; green with MCHR1) and DAPI (blue). Images compare ciliation in cells with or without serum treatment. Bottom, quantification of data for L13-Arl13bGFP, pEGFP-mSmo, and tdTomato-MCHR1-N-10 from 250 cells, from three representative experiments; results following treatment with an inhibitor (alisertib) and a stimulator (ganetespib) of ciliary disassembly are also shown. Data are presented as mean ± SEM, ^∗^*p* ≤ 0.05, ^∗∗^*p* ≤ 0.001, ^∗∗∗^*p* ≤ 0.0001 as compared to control.

Prior to using these models for screening, we evaluated the ciliary localization of the fluorescent marker and utility of the biomarker for measurement of disassembly using conventional low throughput methods. Figure 3 is a confocal image of cell lines selected for each of the three probes, maintained either under conditions of optimized ciliation (48 h starvation in serum free culture medium or Opti-MEM), or 2.5 h after the addition of 25% fetal bovine serum (FBS) to induce ciliary disassembly. All probes localized effectively to the cilium.

### Protocols for Inducing Ciliary Assembly or Disassembly in a 96-Well Microtiter Plate Format

#### Growth Conditions to Induce Ciliation

High throughput screening (HTS) can be used both to identify compounds that induce ciliary disassembly, or block the ciliary disassembly caused by serum-encoded growth factors. In each case, it is important to optimize ciliation in a 96-well plate format. *We note*, because edge effects (differences in growth pattern of cells located near the edge of a well), a 384-well format is generally not appropriate for experiments involving measurement of ciliation due to heterogeneity of growth habit. An additional key element of HTS is that it relies on analysis of a large number of cells to develop statistically meaningful data. Hence, it is necessary to screen at 20×. One factor to consider is that tagging an endogenous protein would be more physiological and help avoid the issue with overexpressed transgenes themselves affecting ciliary stability. However, we have observed that cell lines expressing low levels of the tagged proteins described here (ascertained by Western blot) are difficult or impossible to visualize at 20× (although resolvable at 60×). Hence, use of more than one tagged cell line, or a tagged cell line followed by use of untagged cells in low throughput/high magnification, is recommended.

**FIGURE 4:**
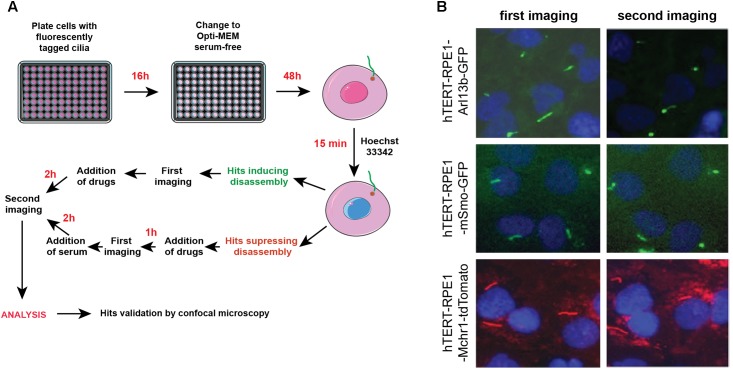
Automated screening workflow and representative images from the ImageXpress microscope. **(A)** Steps for plating, imaging, drug/serum addition, and analysis. **(B)** Epifluorescent images acquired by ImageXpress from first (no serum) and second imaging (after addition of serum control), for the hTERT-RPE1-Arl13b-EGFP (green) hTERT-RPE1-pEGFP-mSMO (green), and hTERT-RPE1-MCHR1-tdTomato (red) models. Note higher background in hTERT-RPE1-MCHR1-tdTomato cells.

Figure 4A,B provide a general experimental schematic and representative images, respectively. For cell plating, bulk reagent dispensers such as the Thermo Scientific Matrix WellMate (Thermo Fisher Scientific, Waltham, MA, United States) can be used. For drug addition, automated liquid handlers can add nanoliter quantities of compounds using pin tools (available from sources such as such as V&P Scientific, San Jose, CA, United States) or liquid dispenser systems [such as the Tecan D300e (Tecan, Switzerland)]. In addition, these steps can be performed manually using hand-held disposable pin tools compatible with a 96-well plate format (for example, from Scinomix, Earth City, MO, United States).

**FIGURE 5:**
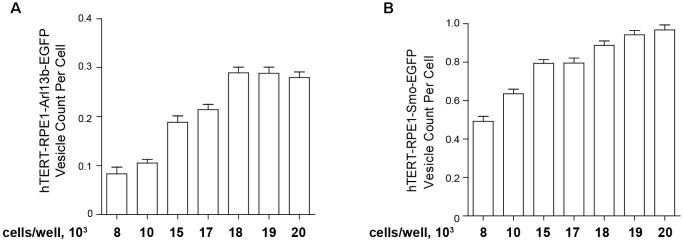
Quantification of ciliation based on plating density. hTERT-RPE1-Arl13b-EGFP **(A)** and hTERT-RPE1-SMO-EGFP **(B)** cells were plated at concentrations shown under conditions described in text, and ciliation assessed after 48 h. Quantitation is based on use of “vesicle total count per cell” in the Transfluor module. Data are presented as mean ± SEM.

For the high throughput assessment of model cell lines bearing fluorescent ciliary tags, for best performance, earlier passage transfected cells (usually p3–p5) are seeded in black 96-well clear bottom microplates (Costar catalog #3603, Corning Inc., Corning, NY, United States) at a set density in 100 μl culture medium (DMEM/F-12 supplemented with 10% FBS, 1× penicillin/streptomycin, 1× Glutamax-I, and 0.01 mg/ml hygromycin B, noted above), then incubated overnight (16 h) in a humidified incubator (37°C and 5% CO_2_). The second day, 100 μl of Opti-MEM medium without FBS and other supplements for 48 h to induce ciliation. This typically leads to robust ciliation (>80%) in these cell models. However, it is important to titrate the initial seeding density. Figure 5 compares the ciliation as a factor of cells initially seeded (from 8000 to 20,000 cells) for the hTERT-Arl13b-EGFP and hTERT-SMO-EGFP cell model; in this case, 15,000–20,000 cells were needed to obtain robust ciliation.

#### Measurement of Drug Effect on Ciliary Dynamics

In general, images of cilia and nuclei are acquired simultaneously, before and after perturbation by serum or drugs. All the steps that require changing of medium or application of drugs can be accomplished using automated liquid handlers or manually, at the discretion of the user.

1. To allow visualization of nuclei as a reference for number of viable cells, medium is replaced with 100 μl of Hoechst-33342 (Life Technologies, Carlsbad, CA, United States) diluted 1:20,000 in Opti-MEM at the beginning of the experiment, and allowed to equilibrate for 15 min at 37°C in 5% CO_2_. At this stage, a first imaging of cilia and nuclei is performed (see next section); these images represent the baseline for quantitation of ciliation prior to perturbation by serum or compounds.
2. To identify compounds that *induce* ciliary disassembly, compounds to be screened are added to the medium. Typically, compounds are prepared as 10 mg/ml stocks in DMSO which can be stored for up to 1 month at -20°C or 3 months at -80°C, then diluted to 1 or 5 μM in DMSO immediately before the experiment. For initial screening for activity in regulating cilia, evaluating drugs using at least two set concentrations between 200 nM and 2.5 μM (final concentration after addition) is a useful range. It is important to include appropriate negative and positive controls. Vehicle (0.01% DMSO) provides a negative control. Serum (25% FBS final volume) provides an essential positive control, which will typically define the maximum level of disassembly. For small molecules, alisertib (MedchemExpress, Monmouth Junction, NJ, United States) and ganetespib (Synta Pharmaceuticals, Bedford, MA, United States) provide useful reference compounds that cause ciliary stabilization and ciliary disassembly, respectively. Typically, compounds are diluted in Opti-MEM and added in a volume of 100 μl per well. *We note*, best results are obtained if care is taken to add compounds to ciliated cells gently (that is, ensuring a low flow rate), to avoid the generation of mechanical shear forces that can independently influence ciliation (Iomini et al., 2004; Delaine-Smith et al., 2014), and to avoid dislodging cells. Cells are then incubated at 37°C in 5% CO_2_ (an exact time should be selected based on initial optimization, then used consistently); subsequent to this incubation, a second set of images is acquired. For the models described here, we typically use 2.5–3 h as the imaging time point.
3. To identify compounds that *inhibit* ciliary disassembly experiment, after the initial imaging (step 1), compounds to be assessed are added immediately with controls in 100 μl Opti-MEM medium without serum (achieving a final volume in the wells of vehicle, 0.01% DMSO; alisertib 200 nM; and ganetespib 500 nM), and incubated at 37°C in 5% CO_2_ for 0.5 h. This initial addition allows drugs time to interact with and inhibit relevant target proteins before initiation of disassembly. After 30 min of pre-treatment with drugs, a first imaging is performed. Subsequently, medium is replaced with 100 μl of Opti-MEM medium containing 25% FBS and drugs at the same concentration used for pre-incubation, and incubated for 2.5–3 h at 37°C in 5% CO_2_. Subsequent to this incubation, a second set of images is acquired.

### Protocols for Imaging Ciliation in High Throughput: Instrumentation, Settings, and Analysis

#### Options for Instrumentation

For the visualization and analysis of primary cilia, we typically use an ImageXpress micro automated microscope equipped with MetaXpress software (Molecular Devices, Sunnyvale, CA, United States). There are a number of alternative instruments available suitable for high throughput, high content imaging studies (reviewed in Buchser et al., 2012). These include the InCELL Analyzer (GE, Chicago, IL, United States) and associated software InCell Developer toolbox (see Dunn et al., 2018); the Thermofisher Cellinsight CX5 and associated Thermofisher HCS Studio Cell Analysis software (Pagliero et al., 2016) (Waltham, MA, United States); and the Operetta and associated Harmony software (Perkin Elmer, Waltham, MA, United States). In addition, any image acquisition technology can be coupled with multiple free image analysis software packages such as ImageJ (Schindelin et al., 2015) and Cell Profiler (Carpenter et al., 2006; Kamentsky et al., 2011).

#### Image Acquisition

Image acquisition is performed on an ImageXpress micro automated imaging system equipped with environmental control (Molecular Devices, Sunnyvale, CA, United States) driven by MetaXpress software. To capture as many cells as possible and to ensure data represent a homogeneous response across sample wells, nine images per well are taken, using two channels to capture matching signal from Hoechst-stained nuclei (DAPI channel, ex 377/50, em 447/60), and cilia tagged with fluorescing probes (ex 472/30 em 520/35 for EGFP, ex 525/40, em 585/40 for tdTomato). Epifluorescence images are acquired with a 20× objective (ELWD Plan Fluor, NA 0.45, WD 7.4), using laser auto-focus with a z-offset. Nuclear images are acquired first, followed by cilia images at a set z-offset from the DAPI channel. The imaging routine was optimized for speed to minimize image acquisition time with live samples. The focus routine allows for one 96-well plate to be imaged in approximately 1 h. During optimization of the image acquisition routine, nine images per well represented the minimum number of images required to provide sufficient ciliated cells per well. Subsequent to this imaging of baseline ciliation, cells receive the next perturbation (serum addition and/or drug addition) and timed incubation prior to a second imaging. Automated imaging crucially allows for the first and second images to represent paired fields.

#### Image Analysis

In the primary analysis approach used for screening, acquired images were analyzed with MetaXpress software (Molecular Devices^1^) using the Transfluor module. The Transfluor module has been used successfully to identify various classes of intracellular vesicles and related signaling events (e.g., Wang et al., 2004; Garippa et al., 2006; Ross et al., 2008). Alternatively, image analysis methods to identify cilia can be developed using settings within the module MultiWavelength Scoring (MWS). As yet another approach, thresholding in ImageJ can be effective, and can be used to filter results for alternative parameters to those reported by a dot counting module such as Transfluor.

**FIGURE 6:**
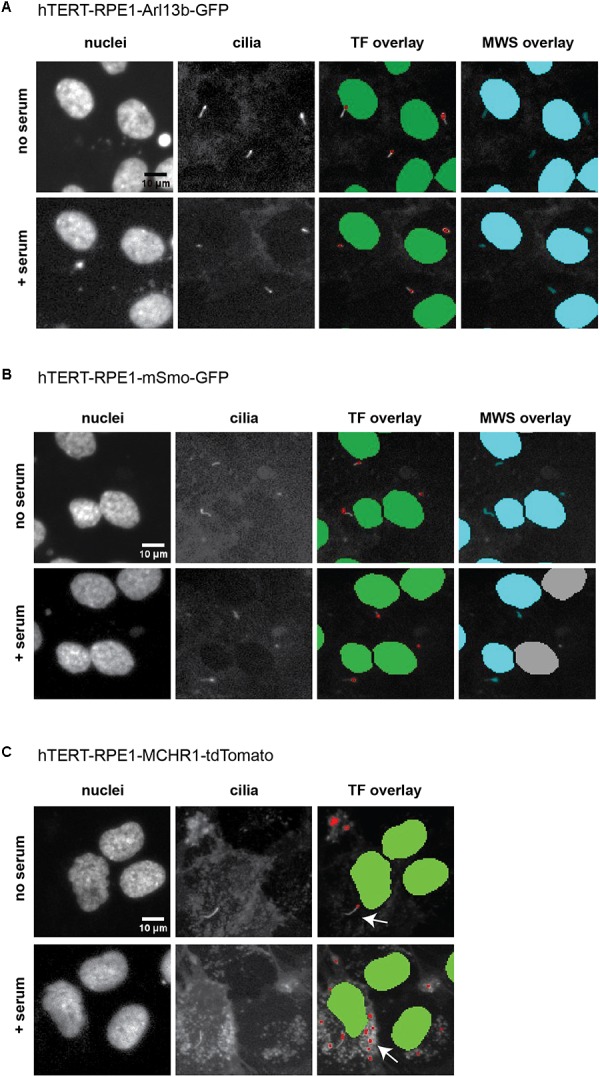
ImageXpress images of cilia, emphasizing thresholding issues. Representative images of the three models of ciliation evaluated in this method, and typical image segmentation by MetaExpress software modules Transfluor (TF) and Multiwavelength Scoring (MWS). **(A)** hTERT-RPE1-Arl13b-EGFP. **(B)** hTERT-RPE1-SMO-EGFP. **(C)** hTERT-RPE1-tdTomato-MCHR1. Left panels show Hoechst stained nuclei, middle panels show labeled cilia, and right panels are representative image segmentation as recognized by the software (shown as an overlay of the cilia image. For TF, green is nuclear segmentation, red is the cilia segmentation; for MWS, nuclei and cilia are overlaid in cyan). For each cell model, no serum and + serum panels represent the same image field. In **A**, settings for both TF and MWS modules can be developed that discriminate between background and cilia; for **B**, settings for TF but not MWS can be used. The tdTomato-MCHR1 model, shown in **C**, has significant intracellular signal, particularly following addition of serum (see arrows in the right panel), which render automated counting unreliable by either TF or MWS approach.

Within the Transfluor module, settings for “vesicle” generate a pixel mask or segmentation overlay that identifies objects within the image as either “nucleus” or “vesicle” (see example overlays in Figure 6). The “vesicle” settings are used to identify cilia using size minimum and maximum criteria in conjunction with intensity thresholds. Determination of appropriate settings for cilia automated counting are determined using the vehicle “no serum” and vehicle “plus serum” controls, to establish the intensity threshold. Minimum and maximum values for vesicle size are dependent on the cell model being utilized, due to varying intensities of the individual fluorescent tag in that cell model. For the three cell models analyzed here, the values for minimum vesicle size were in the range of 1–2 μm while the maximum varied from 8 to 11 μm. Representative images of L13-Arl13bGFP, tdTomato-MCHR1, and pEGFP-SMO expressing cells before and after serum addition, and the accompanying image segmentation overlay, are shown in Figure 6. L13-Arl13bGFP resulted in the best results from this automated analysis, based on the low occurrence of vesicles with GFP fluorescence in the cytoplasm. pEGFP-Smo expressing cells also yielded a signal suitable for statistically significant, consistent quantitation, albeit with a greater level of background signal (Figure 6). In contrast, tdTomato-MCHR1 contained too high a level of background labeled vesicles to be useful in high throughput approach. Hence, the L13-Arl13bGFP and pEGFP-SMO models are used for automated screening, while the tdTomato-MCHR1 models are used for subsequent validation by confocal analysis.

**FIGURE 7:**
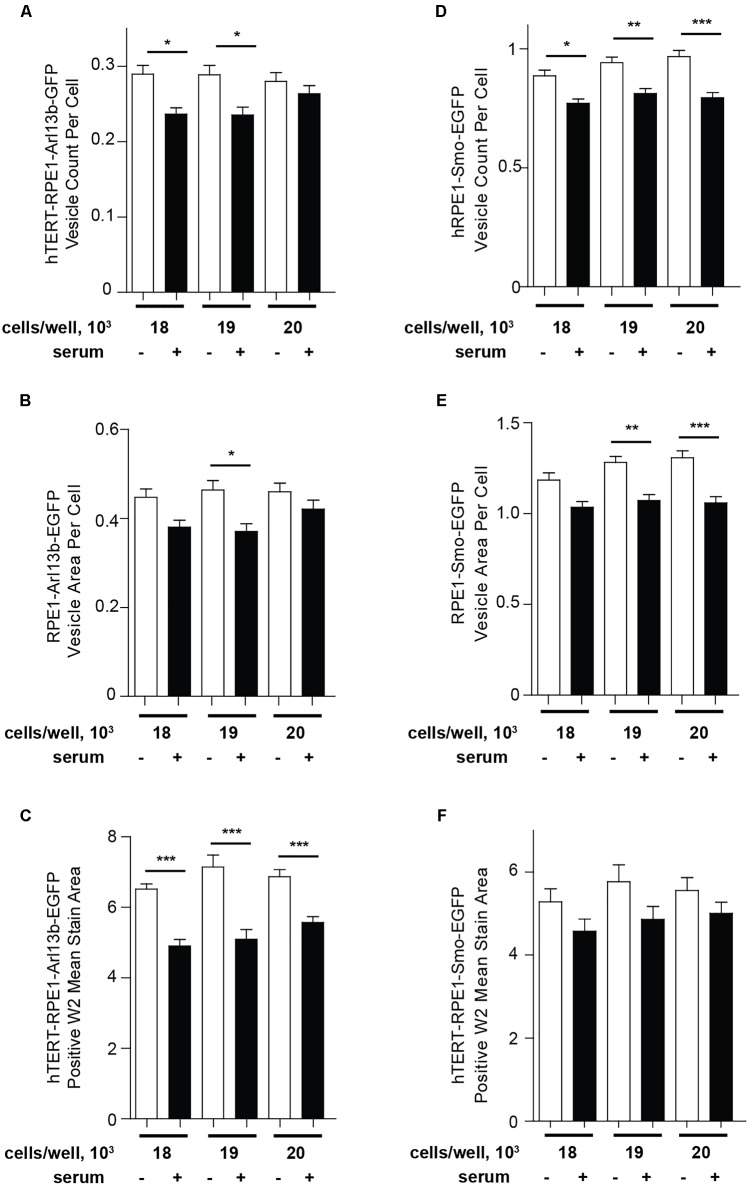
Quantification of ciliation using Transfluor and MultiWavelength Scoring modules. **(A,B)** Analysis of ciliation in hTERT-RPE1-Arl13b-EGFP and hTERT-RPE1-Smo-EGFP cell lines based on “vesicle count per cell” and “vesicle per cell” in the TF module (**A,B** for Arl13b, **D,E** for SMO), and positive W2 mean stain area in the MWS module (**C** for Arl13b, **F** for SMO). Data are presented as mean ± SEM, ^∗^*p* ≤ 0.05, ^∗∗^*p* ≤ 0.001, ^∗∗∗^*p* ≤ 0.0001 as compared to control.

As an alternative to Transfluor, we have also used the MWS module, a standard image thresholding approach is used to segment nuclei utilizing images acquired in the DAPI channel (as in the Transfluor module), and to segment cilia as objects within the images acquired for visualization of cilia. The module for both nuclear segmentation and cilia segmentation requires the researcher to develop settings that successfully identify nuclei and cilia utilizing object minimum size (for cilia in our experiments, this ranges from 1 to 1.8 μm) and maximum size (which varies from 5 to 10 μm). It is also necessary to develop a minimum intensity (settings from 78 to 150 in the MWS module, dependent on the strength of the fluorescence signal) and a minimum stained area (settings from 1.5 to 3). The MWS captures more intracellular background than the Transfluor module, so that in our hands, only the L13-Arl13bGFP model was suitable for use with this approach, due to the low cytoplasmic staining in this line (Figure 6). However, if the cell line in use is suitable, MWS can provide a powerful tool to measure cilia parameters such as “positive stained area” or “% positive W2” (where W2 is wavelength utilized for cilia imaging; and % positive reflects the number of cells per image scoring within the segmentation metrics) allowing a more detailed phenotypic characterization than Transfluor analysis alone, permitting rough inference of ciliary length. As shown in Figure 7, the Transfluor module yielded statistically significant results for serum induced ciliary disassembly in both the L13-Arl13bGFP and pEGFP-SMO models, while the MWS module did so only in the L13-Arl13bGFP cell line.

*It is important to note*, additional parameters can be extracted from the MWS approach using custom software accessories (such as custom module developers, for example, Integrated Morphometry Analysis within MetaXpress). Utilization of these tools with the MWS module, in conjunction with a confocal imaging system, can yield more detailed cilia characteristics such as individual cilia length. A secondary application of the MWS module is to allow filtering of images prior to hit picking; for example, the metric “positive stained area” for the cilia channel can be used to identify images with excessive background; allowing the researcher to identify problematic images that require manual supervision prior to hit-picking.

Results from these types of analyses return multiparameter datasets quantitating each image. The parameters from Transfluor analysis used for this method include “vesicle count per cell” as shown in Figure 5, with cilia number normalized to number of nuclei. Other metrics reported, which can be used for some analyses, include “total vesicle area” and “vesicle area per cell,” as shown in Figure 7. Data from image analysis can be returned to the investigator in two ways: (1) “summary” data representing the data from all nine images calculated as mean or sum and (2) “site” data which reports the metric of interest per image (or “site”). Therefore “summary” data represent a numerical quantitation of metrics at the level of the well, while the “site” data provide the same metric within each image. Importantly for this protocol, the automated imaging systems returns the plate to the same X,Y,Z coordinate, allowing the same fields to be acquired in both imaging runs. This is critical for the evaluation of cilia by this method; as this is an epifluorescence image and the cilia is changing in the Z dimension, the availability of the paired images allows for a critical quality control supervision without which this screening method would not be sufficiently robust to identify wells of interest.

With the Transfluor analysis, the first step in evaluation of wells for perturbation (drug, growth factor, serum) induction of cilia disassembly is to normalize all wells to the mean of all control wells for “vehicle and starved” (representative of maximal ciliation) using the metric “vesicle count per cell” (vesicle count per cell *sample* ^SUMMARY^ ^MEANIR2^/vesicle count per cell *control* ^SUMMARY MEANIR1^; where IR1 = image run1 or baseline and IR2 = image run 2 or post-perturbation). It is important to note that using an automated system based on epifluorescence, the change in ciliation rate induced by a strong stimulus such as 25% serum is in the range of ∼20–60%, compressing the dynamic range from that detectable by confocal microscopy. However, the ability to image large numbers of cells nevertheless results in highly significant data. In scale with this compressed dynamic range, small molecule hits of interest often first appear as differences of ∼10%, based on mean values; greater differences are detected in follow-up experiments, after optimization of dosing range and use of confocal scoring systems.

The second step, performed after this initial cutoff, is to review individually the nine imaged primary hit wells via comparison of individual paired images for image run 1 and image run 2 (total vesicle area *sample*^SITEIR2^/total vesicle area *sample* ^SITEIR1^). This step allows the elimination of any “outlier images” including images that showed significant loss of cells or poor image quality. This typically improves the screening metric. Processing the resultant data allows selection of the two possible classes of ranked “screening hits” (for enhanced disassembly, or for resistance to disassembly). It is important to note that screen conditions must be optimized for either identification of cilia disassembly or resistance to disassembly; these hits are not identified concurrently in the same screen. The results are evaluated using standard statistical methods, and the significance usually is determined using *p*-values.

### Confirmation of Cilia-Targeting Ability of Compounds Using Confocal Microscopy

All HTS approaches are useful for screening large numbers of specimens quickly, but are not ideal for generating high resolution data. Further, prudence in assigning activity to candidate hits requires measurement of effect using an alternative assay system to prioritize follow-up experiments. Although the screens described above are performed using duplicate wells for technical repetition, and at least two independent runs for biological repetition, before hit selection, we routinely confirm cilium-modulating activities of candidate drugs using a confocal fluorescence microscopy approach. In general, immunofluorescence protocols for analyzing cilia are well established, and have been used by many groups. For the novice, several facts are important to note. First if it is intended to use a fluorescent protein as one of the markers for assessment in fixed cells, it is important to avoid methanol as a fixation method, as it will greatly reduce signal. Second, combined analysis of a probe localized to the cilium with a second probe localized to the basal body is extremely helpful in clarifying true from false positives. Third, one way in which a fixation-based protocol differs from the automated live-imaging approach is that imaging can only be performed at the end of the experiment. Hence, it is essential to perform sufficient controls to provide comparison sets for maximal and minimal ciliation under the conditions of the screen.

#### Protocol for Confirmation of Ciliation-Regulating Drug Hits by Standard Immunofluorescence

1. Cell lines bearing fluorescent ciliary marker proteins (RPE1-Arl13b-EGFP, RPE1-SMO-EGFP, or RPE1-MCHR1-tdTomato) are plated in 1 ml culture medium (DMEM/F-12) in a 24-well plate in which each well contains one sterile coverslip pre-coated with collagen (StemCell Technologies, Vancouver, BC, Canada). As with HTS, it is useful to perform preliminary tests to establish optimal plating density; we have found a density of 25,000 cells/well is typically appropriate. Incubate cells overnight (16 h) in a humidified incubator (37°C and 5% CO_2_) to allow cells to attach tightly to the cover slips, and attain ∼60–70% confluency.
2. The second day, replace medium with Opti-MEM lacking serum, and incubate for another 48 h at 37°C and 5% CO_2_. At this point, visual inspection of the plate by light microscope should indicate that cells are 90–100% confluent, but not overgrown. At this point, perform treatments with drug, control, and serum addition comparable to those used in HTS but omitting the step of Hoechst dye addition, scaled up to the larger medium volume.
3. At the experimental endpoint (∼2.5 h after the addition of drugs if measuring induced disassembly, or the addition of serum if measuring blockade of disassembly), fix cells with 250 μl 4% PBS-based PFA for 7 min. Next, remove PFA, wash one time in 1 ml PBS and add 250 μl 0.1% Triton X-100 in PBS for 10 min to permeabilize cells. Wash one time with 1 ml PBS and proceed to blocking.
4. Add PBS-based 3% bovine serum albumin solution for 30 min to block non-specific antibody interactions. Incubate cells with selected antibodies using standard protocols optimized for each reagent for immunofluorescence by the manufacturer^2^. In the human cell lines described here, useful primary antibodies for visualizing the ciliary basal body include rabbit anti-pericentrin (1:200 dilution; IHC-00264, Bethyl Laboratories, Inc., Montgomery, TX, United States) or anti-γ-tubulin (1:200 dilution; T5192, rabbit; Sigma–Aldrich Corp., St. Louis, MO, United States). The mouse anti-acetylated-α-tubulin (1:200 dilution; sc-23950, Santa Cruz Biotechnology, Inc., Santa Cruz, CA, United States) can be used for cilia visualization. Secondary antibodies include anti-rabbit conjugated to Alexa Fluor 488 or Alexa Fluor 568 (1:2000 dilution, Life Technologies, Carlsbad, CA, United States). Typical time of antibody incubation is 1 h at room temperature for primary, and 1 h at room temperature for secondary (with incubations performed in the dark), with three washes in PBS after each incubation. After the final wash, coverslips are removed from a plate and mounted using Prolong Gold with DAPI (Life Technologies, Carlsbad, CA, United States) to also visualize DNA.
5. All standard confocal microscope systems can be used to visualize cilia. In our hands, we image samples using the Leica SP8 advanced confocal system. Typically, we count 250 cells per condition at 63× magnification. Images were obtained by Leica Application Suite Advanced Fluorescence (LAS AF) software. An important issue to consider when imaging is the cell density; ideally, there should be 50–60 cells evenly distributed cells in the visual field when using these settings. Significant deviations from this number will increase variability due to overgrowth (uneven disassembly) or low density (low initial frequency of ciliation).
6. Hit confirmation of the phenotype scored in the screening environment is determined by a *p*-value determined by *t*-test to examine extent of ciliary loss, from quantitation of confocal images. Agents that were validated by these criteria were considered validated hits, and taken for further functional characterization.
7. *We note*, although both the RPE1-Arl13b-EGFP and RPE1-SMO-EGFP models are suitable for statistically significant quantitation using the methods described, RPE1-MCHR1-tdTomato model shows an accurate results only when analyzed using a low throughput confocal system, at higher magnification. Using two models for preliminary screening and one or more alternative model for confirmation has some advantages; most notably that it would eliminate hypothetical screening artifacts linked to drug effects on the Arl13b-EGFP or SMO-EGFP ciliary probes, by interfering with the biological regulation of Arl13b or SMO.

## Discussion

Because many genetic and sporadic diseases are now known to involve changes in ciliation, and because a growing number of drugs are appreciated as affecting ciliary structure and function, there is clear value in developing efficient tools and methodologies for high throughput profiling of ciliary phenotypes. In the method described here, we have used a live-cell imaging approach utilizing freely available fluorescent-cilia fusion proteins. As the methods and representative data above indicate, there are strengths and weaknesses associated with the use of fluorescent cilia-targeted proteins as a HTS modality to assess drug activity. As strengths, once a cell line with an appropriate cilia-localized protein has been developed, screening involves a very limited number of steps, and costs of reagents are low, allowing the facile assessment of a large number of compounds. Data are read automatically, and quantified, removing potential sources of experimental bias. Multiple distinct modules are available for imaging systems, allowing the screener to adjust sensitivity level, and include or exclude partial phenotypes. Hits identified by this approach can rapidly be validated using an orthogonal fixation-based method, using the same or alternative ciliary proteins for antibody-based visualization.

Based on extensive practical experience, there are some limitations of HTS methods, which vary from model to model. As noted above, in some cases, the over-expressed tagged protein may be suitable for confocal analysis, where the *z*-axis can be readily modified and limited in depth; or for live cell imaging, but not for automatic counting, which is typically based on epifluorescence, and where it is impossible to exclude competing signal from the cytoplasm. Among the examples we test, the RPE1-MCHR1-tdTomato cell line has significant interfering background signal, and is not appropriate for high throughput analysis, although is suitable for confocal assessment. However, by carefully matching image analysis tool with cell line model, robust results can be achieved. As a particular strength, by utilizing biological and technical controls and development of appropriate settings in image analysis software, large number of images can be screened with the approach described herein. For automated approaches to be most effective, prior to commencement of large screening, it is important for researchers to invest the time at the beginning of experimentation to ensure that each metric returned by these software tools is validated by statistical analyses and manual supervision of images.

When using automated approaches for compound screening, it is important to be aware of potential confounding issues. One common issue is the inherent fluorescence of some compounds, which can affect the assay and final analysis. In our hands, some drugs we have worked with have a strong yellow color, which caused higher background in the GFP channel on the ImageXpress; such competing signal from compounds can compromise the use of automated image analysis. An additional technical issue to consider is the possibility that over-expressed autofluorescent proteins that localize in part or in sum to cilia may affect ciliary integrity and rate of resorption. For example, in our hands, cilia in the RPE1-Arl13b-EGFP cell line resorb at 3 h after treatment with serum, in contrast to the hTERT-RPE1 parental control, where resorption occurs at 2 h after treatment. In practice, this is a minor feature of screening that can be easily accommodated by adjustment of assay times. In principle, this may mean the expressed protein is affecting ciliary trafficking machinery in a manner that alters the spectrum of which drugs do or do not impact ciliation. If resources are available, such issues can be eliminated by performing parallel screening in two independent cell lines expressing different ciliary tags that have distinct biological activities. Whether or not parallel screening is performed, is important to confirm the activity of “hit” proteins during the validation process, in multiple cell lines lacking overexpressed ciliary proteins.

The protocols described above focus on the identification of small molecule compounds that regulate ciliary disassembly. It is also possible to study compounds that regulate ciliary assembly, using modifications of the described protocols. However, identification of such compounds is more challenging, because of several technical and biological considerations. Ciliary disassembly is performed over a short time period (2–3 h) in cells pre-synchronized by contact inhibition and serum starvation into quiescence. Ciliary assembly occurs over 24–72 h (depending on the cell model), in cell populations that are initially asynchronously growing. Hence, drugs to be assessed must be added at early time points, and maintained in the culture medium for at least 24 h. As a result, there is much more opportunity for “reduction in ciliation” due to confounding alternative drug activities to be made manifest. Drug actions resulting in non-specific reduction of ciliation would include general cell cycle arrest at a stage where cilia are already resorbed, induction of apoptosis or other forms of cell death, change in cell-cell attachment properties (so contact inhibition does not occur), and detachment from the plate, among others. Considerable care must be taken in designing controls if attempting such experiments.

The three model cell lines described here – RPE1-Arl13b-EGFP, RPE1-SMO-EGFP, and RPE1-MCHR1-tdTomato – use cilia-localization sequences provided by three very well-studied ciliary proteins that associate with the ciliary membrane. Over the past several years, large scale studies have defined the proteome of the ciliary membrane, axoneme, basal body, and associated structures (Ishikawa et al., 2012; Mick et al., 2015; Dean et al., 2016; Kohli et al., 2017). These studies provide a useful resource for identifying alternative ciliary tags, some of which can illuminate specific ciliary domains such as the ciliary tip or the ciliary transition zone. We view the use of autofluorescent visualization proteins associated with the ciliary membrane as likely to yield less problematic outcomes (e.g., disruption of ciliary transport) than those targeting core structures such as the IFT machinery, but for any new system, empirical assessment will be needed. Finally, other parameters of ciliary dynamics, such as ciliary beat frequencies for motile cilia (beyond the scope of this methods study) have been obtained through moderate-throughput, computational analysis of videos (Mantovani et al., 2010). Fluorescent tags can similarly augment such approaches.

## Author Contributions

PZ, AK, and VK contributed to experimental data. ME assisted during HTS procedures including development and execution of image acquisition and image analysis routines. EG and HL supervised the staff and ensured the quality of data analysis. PZ, AK, EG, and ME wrote and edited the manuscript.

## Conflict of Interest Statement

The authors declare that the research was conducted in the absence of any commercial or financial relationships that could be construed as a potential conflict of interest.

## Footnotes

1 www.moleculardevices.com

2 https://www.abcam.com/protocols/immunocytochemistry-immunofluorescence-protocol
